# Proteomic characterization of hydatid cyst fluid: two-dimensional electrophoresis (2-DE) setup through optimizing protein extraction

**DOI:** 10.1186/s13104-020-05433-3

**Published:** 2021-01-11

**Authors:** Bahareh Sedaghat, Homa Hajjaran, Fatemeh Sadat Sadjjadi, Soudabeh Heidari, Seyed Mahmoud Sadjjadi

**Affiliations:** 1 Department of Parasitology and Mycology, School of Medicine, Shiraz University of Medical Sciences, Shiraz, Iran; 2 Department of Medical Parasitology and Mycology, School of Public Health, Tehran University of Medical Sciences, Tehran, Iran; 3 Faculty of Paramedical Sciences, Proteomics Research Center, Shahid Beheshti University of Medical Sciences, Tehran, Iran

**Keywords:** *Echinococcus*, Hydatid cyst, 2DE, Proteomics, Clean-up

## Abstract

**Objective:**

Proper characterization of hydatid cyst fluid (HCF) is useful for diagnostic and follow up purposes of cystic echinococcosis/hydatidosis, which is an important zoonotic disease. In this regard, proteomics methods are very helpful. The present study was conducted to compare three protein extraction methods for HCF collected from sheep liver hydatid cysts including, trichloracetic acid (TCA)/Acetone precipitation, TCA/Acetone along with dialysis, and combination of 2-D Clean-up Kit and dialysis followed by two-dimensional electrophoresis (2-DE), to achieve better resolution in the proteomic characterization of HCF proteins.

**Results:**

The 2-DE of TCA/Acetone products showed a lot of smears in the background of gels; TCA/Acetone with dialysis showed greatly reduced smears while the 2-D Clean-up Kit together with dialysis showed sharp spots and least smears. Three-dimensional images of separated spots created by Progenesis SameSpots software showed the best result was achieved by 2-D Clean-up Kit and dialysis.

## Introduction

Cystic echinococcosis (CE)/hydatidosis has a worldwide distribution. Infection with the larval stage of *Echinococcus granulosus* sensu stricto, as the causative agent of CE, in the intermediate hosts cause the development of hydatid cyst in different organs, mainly in the liver and lungs [1–4].

*Echinococcus granulosus* is an emergent or re-emergent human zoonosis and listed as one of the 17 neglected tropical diseases by the World Health Organization [3–6].

Hydatid cyst fluid (HCF) has been a good source of proteins as biomarkers for immune-diagnosis of the disease [7–9]. Different methods have been used for protein extraction from HCF [8, 10]. However, its cross-reaction with proteins overexpressed in other diseases reduces the specificity of current antigens. Hence, new antigens are needed for serodiagnosis of CE [8].

In this regard, proteomics as a new and powerful method can be used for better characterization of HCF proteins for diagnostic and prognostic purposes [11]. Several studies have attempted to examine the parasite protein in HCF and characterize the proteins [11–15].

For the first time Chemale et al. [12] attempted to analyze the cattle HCF proteins, but were unsuccessful to establish a two-dimensional electrophoresis (2-DE) database.

2-DE is one of the most popular techniques used in the field of proteomics. Attempts have been made to use proteomics as a practical method for diagnosis of various diseases including CE [16, 17].

However, the large amount of proteins especially albumin and globulins, salt, other small ionic components, polysaccharide, and lipid in body fluid cause horizontal and vertical streaking problems in the 2-DE method [12, 18, 19].

The 2-DE result mostly depends on the selection of an effective method of sample preparation for protein extraction [20]. This can significantly affect the isoelectric focusing (IEF) in the first dimension. In many cases, protein extraction from samples is complicated due to the existence of non-protein contaminants [21]. Regarding the above problems, the present study is going to compare different practical methods for optimizing protein extraction and 2-DE techniques to achieve better resolution in the 2-DE gel for proteomic characterization of HCF.

## Main text

### Methods

#### Sample collection

HCF was collected from infected sheep liver hydatid cysts slaughtered in Shiraz slaughterhouse, Southern, Iran. The protoscoleces and other debris were removed from the aspirated HCF by centrifugation at 10,000*g*, for 15 min at 4 °C and clear HCF stored at − 80 °C prior to use. A protease inhibitor cocktail (25 mL per tablet; Roche, Germany) was added to inhibit protein degeneration of HCF.

#### Protein sample preparation for 2-DE

Proteome preparation was made using three methods as follows:

#### TCA/Acetone precipitation

**Fig. 1:**
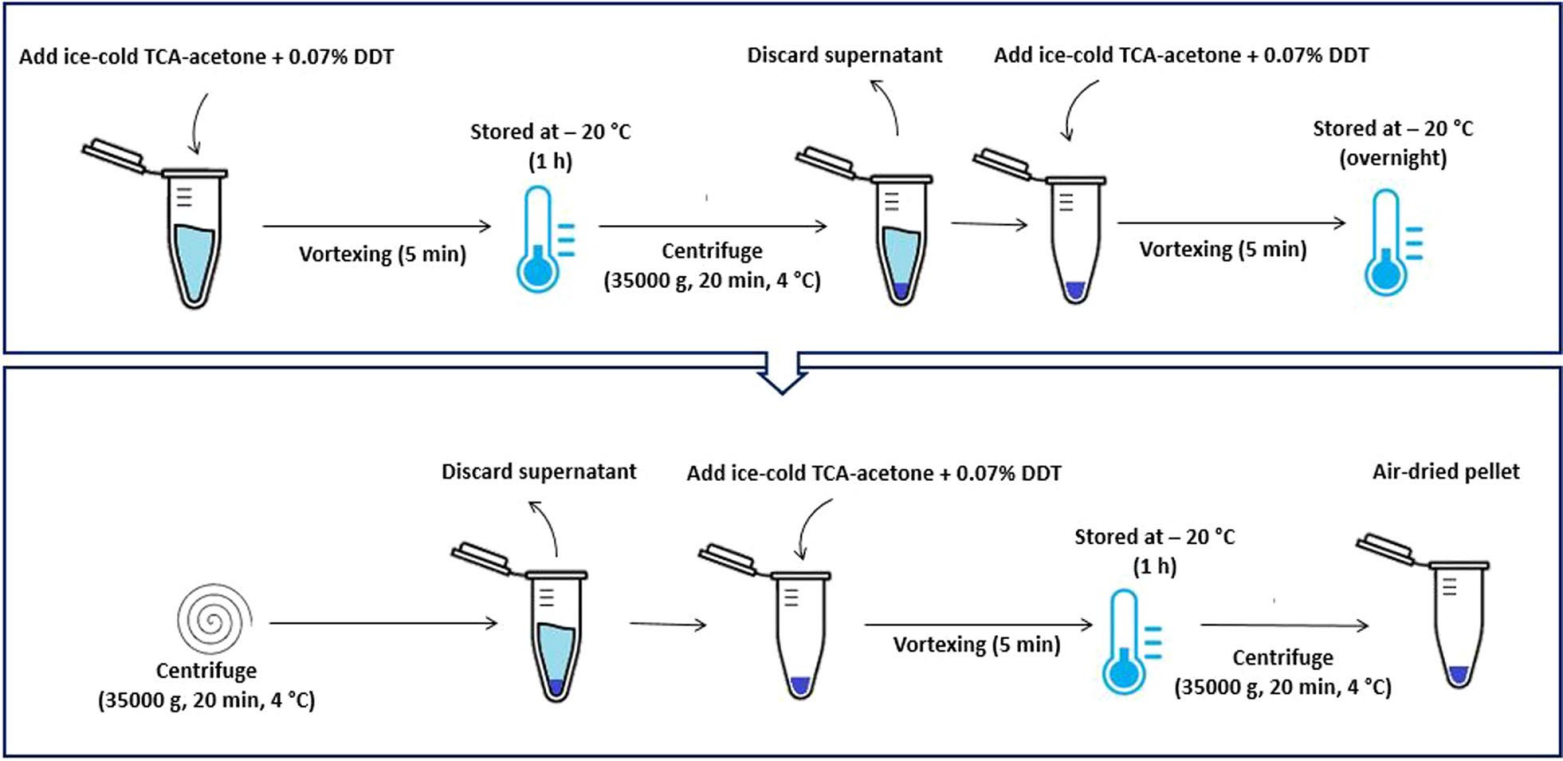
A schematic pictorial laboratory algorithm of the TCA/Acetone precipitation protocol. *TCA* trichloracetic acid, *DTT* dithiothreitol

In this technique [22], 500 μL HCF was transferred into 1.5 mL microtubes with 1000 μL of ice‐cold acetone containing 10% of trichloracetic acid (TCA) and 0.07% dithiothreitol (DTT). The samples were incubated 1 h at − 20 °C and vortexed for 5 min followed by centrifugation at 4 °C for 20 min at 35,000*g*. The supernatant was discarded and the pellet was re-suspended in 1.8 mL of ice-cold acetone containing 0.07% DTT, vortexed for 5 min, and was incubated overnight at − 20 °C. The specimen then was centrifuged at 35,000*g* at 4 °C centrifuge for 20 min followed by removing the supernatant. The pellets were then washed with cooled acetone containing 0.07% DTT and were incubated for 1 h at − 20 °C and were vortexed for 5 min. This step was repeated twice. Finally, the pellet was air-dried and stored at − 80 °C before use (Fig. 1).

#### Dialysis with TCA/Acetone

In this approach, the HCF must first be purified before using the TCA/Acetone method. An Amicon (cut off: 3KDa, Amicon Millipore, USA) unit was used for filtration, desalting, and other pollution using double distilled water. This also increased the concentration of HCF up to 5 times.

#### 2-D Clean-up Kit with dialysis

In this part, 2-D Clean-up Kit (BIO-RAD) together with dialysis was used to clean-up and purify the HCF. The 2-D Clean-up Kit has five regents including precipitation agent 1, precipitation agent 2, wash reagent 1, wash reagent 2 and wash 2. Briefly, a total of 500 µg of protein in a final volume of 100 µL was transferred into a 1.5 mL microcentrifuge tube followed by adding 300 µL precipitating agent 1 and then several times adding solutions, vortexing and centrifuging according to manufacture procedure to yield a pellet which was air-dried at room temperature for 5 min for further use. The entire process can be completed in approximately one hour.

### 2-DE

#### Rehydration step

The protein pellet was solubilized into an immobilized pH gradient (IPG) rehydration buffer containing 7 M urea, 2 M Thiourea, 2% CHAPS, 50 mM DTT, 0.5% Bio-Lyte ampholyte (pH 3–10) (Bio-Rad, USA), 0.001% bromophenol blue. The total protein concentration of each sample was determined using the Bradford assay (Bio-Rad, USA) [23]. Rehydration volume for the linear IPG strip 7-cm (pH 4–7, Bio-Rad) was 125 μL which contains 25 μL/mL protein concentration. IPG strips were passively rehydrated in a tray for 18 h while mineral oil was added to prevent evaporation.

#### IEF using the desalting program

The following steps were made for IEF: Step 1: 50 V (linear) for 4 h; step 2: 250 V (linear) for 1 h; step 3: 1000 V (linear) for 1 h; step 4: 2000 V (linear) for 1 h; step 5: 4000 V (linear) for 2 h; step 6: 4000 V (rapid) for a total of 13000Vh for the entire run. IEF was run using a Protean IEF cell (Bio-Rad).

#### Equilibration and SDS-PAGE step

Following IEF, the strips were reduced in equilibration buffer I containing 6 M urea, 87% glycerol, 2% SDS, 0.375 M Tris, (pH = 8.8), 0.002% bromophenol blue, and 1% DTT over 15 min while shaking.

Equilibration buffer I was discarded and replaced with equilibration buffer II containing 6 M urea, 87% glycerol, 2% SDS, 0.375 M Tris (pH = 8.8), 0.002% bromophenol blue, and 2.5% iodoacetamide over 15 min while shaking.

The IPG strip was loaded onto an SDS PAGE gel composed of a 12% separation gel followed by proteins staining using silver nitrate.

### Results

**Fig. 2:**
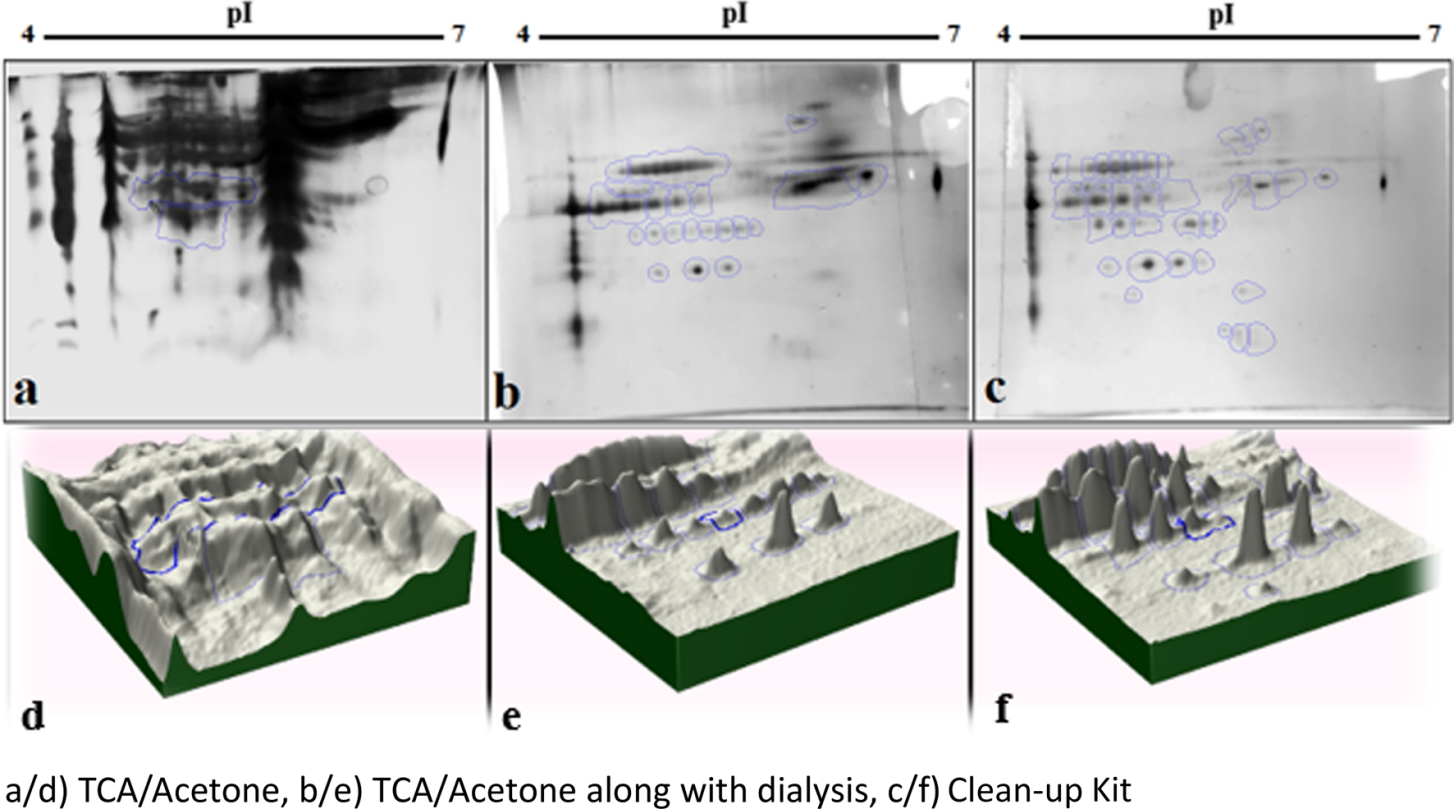
2-DE scaned images of extracted proteins from sheep HCF samples using three methods: **a** TCA/Acetone, **b** TCA/Acetone along with dialysis, **c** 2-D Clean-up Kit along with dialysis. **d**–**f** Three dimensional images of separated spots created by Progenesis SameSpots software. Each peak is representative of each spot

The characteristics of each method and the results obtained from them are summarized in Table 1. The 2-DE map resulted from TCA/Acetone method showed indiscernible protein spots with no clear boundary between them as well as a lot of smears in the gel background (Fig. 2a). The gel showed considerable horizontal and vertical streaking, which made it difficult to judge the TCA/Acetone result. The 2-DE map using TCA/Acetone combination with dialysis (Fig. 2b), showed a better resolution of background such that smears and impurities were greatly reduced, protein spots were separated such that horizontal and vertical streaking was greatly reduced.

**Table 1:** Comparison of the TCA/Acetone, TCA/Acetone with dialysis and, Clean-up Kit methods with dialysis for protein precipitation of hydatid cyst fluid (HCF) based on their outlay and quality results

Methods	Laboratory materials and facilities	Outlay	Quality	Outcome
TCA/Acetone	Acetone, TCA, DTT	Low	Poor	A lot of smears and indiscernible protein spots
TCA/Acetone + dialysis	Acetone, TCA, DTT 3 kDa centrifugal filter (Ultra-0.5)	Middle	Good	Fewer smears and separated protein spots
2-D Clean-Up Kit + dialysis	2-D Clean-Up Kit 3 kDa centrifugal filter (Ultra-0.5)	High	Best	Without smears and sharp and clear protein spots

*TCA* trichloracetic acid, *DDT* dithiothreitol

The 2-DE map using the 2-D Clean-up Kit (Fig. 2c), showed the best result and almost all impurities were removed such that the protein spots were clearer and the separation of proteins was well done.

The protein expression changes between groups were determined by Progenesis SameSpots software (Non-Linear Dynamics, Newcastle-Upon-Tyne, UK). Using the software, the gels were analyzed in several steps including quality control of gels, comparing the processed gels, detecting protein spots, evaluating the color intensity of spots, spots alignment, and statistical analysis. Moreover, three-dimensional images of separated spots as a peak were created; such that each peak is representative of a sharp spot in the form of three-dimensional configuration (Fig. 2d–f).

### Discussion

Different methods have been used for parasites proteins analysis during the past decade [8]; of which 2-DE have been reported as the most effective method to characterize the proteins [16].

Since the most important step in any proteomics study is the extraction and preparation of the sample, we focused on optimizing protein extraction from HCF to its proteomic characterization.

As, the first-dimension of 2-DE is sensitive to low molecular weight ionic, salt,and non-protein impurities in the sample; different approaches were used in our work of which a combination of 2-D Clean-up Kit with dialysis, affected protein separation in better quality such that reduced and further improved the quality visualization of the 2-DE gel result happened. As the sample preparation process is important factor for the resolution of the 2D gel and retrieval of protein, it is critical to removal of salts and impurities and enrichment of the proteins [21]. In this regard, different desalting and removal impurities methods include centrifugal filter devices, dialysis, protein precipitation, Bio-Spin column, and use of 2-D Clean-up Kits have been described [20, 24].

Yuan et al. [21], used desalination for cerebrospinal fluid (CSF) proteome preparation by four different methods. They concluded that due to high salt and low protein concentration in CSF, the highest protein recovery and desalination were achieved with Bio-Spin column, followed by ultrafiltration method [21].

Thus, the TCA/acetone method greatly reduces lipids, polysaccharides, salt, and other impurities in body fluid [20], but this method alone is not enough, as demonstrated in this study TCA/Acetone method alone showed horizontal and vertical streaking, The analysis of HCF proteins of cattle by adding TCA for precipitation yielded a better purification; however, due to the effect of highly abundant albumin and immunoglobulin, the establishment of a 2-DE database was unsuccessful [12]. On the other hand, Islam et al. [25] demonstrated that performed 10% TCA alone is not sufficient to remove contaminants in the plant.

The quality of the map of 2-DE gel of HCF in this study was similar to the results of Ahn et al. [13] who concluded an almost good resolution map and the spots were clearly visible.

The map of the 2-DE gel of protoscoleces by Hidalgo et al. [26] had a bit of smear but the spots were clearly visible. The map of the 2-DE gel of HCF in our study was in contrast to the study of Li et al. [9, 27], who used a 2-D Clean-up Kit, the number of spots was very low and the map was very blurry. It could be due to the application of the Aurum serum protein kit. It seems that the kits that eliminate albumin and IgG from serum or plasma samples are not suitable for HCF and may cause major destruction of proteins. There are many commercial albumin removal kits, which due to nonspecific binding, may destroy proteins other than albumin [24]. So, the albumin kit was not applied in our study.

On the other hand, a 2-D Clean-up Kit in combination with dialysis can be used to remove contaminates constituents and improve the 2-DE electrophoresis pattern [24]. So, 2-D Clean-up Kit was used in our study which resulted in more acceptable results. Anyhow, the TCA/Acetone in combination with dialysis which is a low-cost method comparing to the application of commercial 2-D Clean-up Kit yielded a better result.

During the practical work, the paper wicks (Electrode wicks, Bio-Rad) were soaked with water to absorb the salts; however, using these alone was not enough. The first step run by applying low voltage up to a few hours to desalting of the sample was more effective as has been used by Gorg et al. [24].

The desalting IEF program which was used in our study, facilitated achieving a better result. Although various or somewhat similar programs have been used in other CE proteomics studies. In this study, the duration of the first step of the desalination program was set at 4 h by applying low voltage, in the study of Remy et al. [28] the first stage was set at 9 h.

### Conclusion

Our finding demonstrates that the best method for proteomic characterization of HCF isolated from sheep is the 2-D Clean-up Kit along with dialysis, which produces high-quality gel as the outcome. Although the TCA/Acetone and TCA/Acetone with dialysis methods are less expensive than kits; however, considering the production of gels without smears, sharp and clear protein spots in the 2-D Clean-up Kit method, makes it more applicable for further works including mass spectrometry.

### Limitations

Despite the valuable data provided by this study, several limitations affected its findings, including (a) specimen collection and finding a fertile cyst, and (b) need of cold chains for specimens transfer.

## Data Availability

All data generated or analyzed during this study are included in this published article. The original datasets are available upon request to the corresponding author.

## Abbreviations

CE: Cystic echinococcosis
HCF: Hydatid cyst fluid
2-DE: Two-dimensional electrophoresis
IEF: Isoelectric focusing
pI: Isoelectric point
TCA: Trichloracetic acid
DTT: Dithiothreitol
